# Ferromagnetic Phase in Nonequilibrium Quantum Dots

**DOI:** 10.1038/s41598-017-18440-5

**Published:** 2017-12-22

**Authors:** WenJie Hou, YuanDong Wang, JianHua Wei, YiJing Yan

**Affiliations:** 1 Department of Physics, Renmin University of China, Beijing, 100872 China; 2 Hefei National Laboratory for Physical Sciences at the Microscale and iChEM (Collaborative Innovation Center of Chemistry for Energy Materials), University of Science and Technology of China, Hefei, Anhui 230026 China

## Abstract

By nonperturbatively solving the nonequilibrium Anderson two-impurity model with the hierarchical equations of motion approach, we report a robust ferromagnetic (FM) phase in series-coupled double quantum dots, which can suppress the antiferromagnetic (AFM) phase and dominate the phase diagram at finite bias and detuning energy in the strongly correlated limit. The FM exchange interaction origins from the passive parallel spin arrangement caused by the Pauli exclusion principle during the electrons transport. At very low temperature, the Kondo screening of the magnetic moment in the FM phase induces some nonequilibrium Kondo effects in magnetic susceptibility, spectral functions and current. In the weakly correlated limit, the AFM phase is found still stable, therefore, a magnetic-field-free internal control of spin states can be expected through the continuous FM–AFM phase transition.

## Introduction

**Figure 1:**
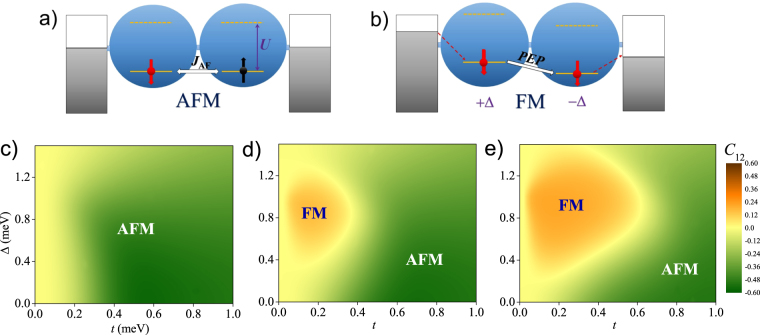
(**a**) Schematic diagram of antiferromagnetic (AFM) state in series-coupled double quantum dots (SDQDs) under equilibrium conditions. *U* is the on-dot electron-electron (*e* − *e*) interaction. *J*_AF_ is the strength of AFM exchange interaction. (**b**) Schematic diagram of ferromagnetic (FM) state in SDQDs under nonequilibrium conditions at finite bias *V* and detuning energy 2Δ. PEP denotes the Pauli exclusion principle during the electrons transport. (**c**–**e**) Magnetic phase diagrams of SDQDs in the Δ − *t* (*t* being the inter-dot coupling) plane by showing the spin-spin correlation function ${C}_{12}\equiv \langle {\overrightarrow{S}}_{1}\cdot {\overrightarrow{S}}_{2}\rangle -\langle {\overrightarrow{S}}_{1}\rangle \cdot \langle {\overrightarrow{S}}_{2}\rangle$ at various bias, (**c**) *V* = 0; (**d**) *V* = 0.5 mV and (**e**) *V* = 1.0 mV.

The ferromagnetism intrinsically origins from the spin-independent Coulomb interaction and the Pauli exclusion principle (PEP), as initially proposed by Heisenberg^1^. The Hubbard model^2^, which includes both two elements with on-site electron-electron (*e* − *e*) interaction *U*, is regarded as the minimal model for ferromagnetic (FM) states. Unfortunately, it has not been well addressed whether the Hubbard model has a general FM phase, except under some special conditions^3–6^. The Hartree-Fock approximation once predicted an itinerant Stoner-like FM phase^7^, but we now know that the mean-field theory deduces incorrect results and the FM region has been overestimated^3^. Besides the Hubbard model, the Anderson (multi)-impurity model^8^ may act as another minimal model for magnetic phase in a bottom-up fashion, with the advantage of implementation simplicity in quantum dots (QDs). For example, the antiferromagnetic (AFM) correlation *J*_AF_ due to nearest-neighbour electron hopping or tunneling *t* (*J*_AF_ ~ 4*t* ^2^) has been well understood experimentally in series-coupled double QDs (SDQDs) [see Fig. 1(a)]^9^. Theoretically, *J*_AF_ is responsible for the AFM ground state at half filling in the Hubbard model, while it induces the spin singlet competing with the Kondo singlet at temperature *T* < *T*_*K*_ (*T*_*K*_ being the Kondo temperature) in the Anderson two-impurity model^9–13^.

Does there exist a FM phase in the Anderson two-impurity model or in SDQDs? That issue may help to understand Heisenberg’s original idea and to determine the FM phase in various strongly correlated models. Please be noted that the sign-indefinite Ruderman-Kittel-Kasuya-Yosida (RKKY) magnetic order, whose implementation must through a third mediated dot in experiments^14^, is not our concern here. What we are seeking is a stable FM phase strong enough to compete with the AFM one in SDQDs, which has not been explicitly determined yet in the phase diagrams of SDQDs^9^ and other two-impurity systems^15^.

The FM phase in SDQDs also has great application potential in solid-state quantum computing. QDs-based spin qubit is one of the most possible physical realization of scalable qubit put forward so far, which has been extensively studied in last two decades^16,17^ since its original proposal in SDQDs^18^. It has the advantages of fast operation and long coherence times but the disadvantage of seriously dependence on magnetic fields. The technical difficulties caused by magnetic fields are transparent: (*i*) The localized oscillating magnetic fields required in qubit or quantum gate manipulation are very hard to realize in practice; (*ii*) The Zeeman energy is an inefficient way to control spin states; and (*iii*) The magnetic fields are incompatible with present large-scale integrated circuit. If a stable FM phase in SDQDs does exist, these difficulties may be overcome by possible magnetic-field-free manipulations.

In the present work, by nonperturbatively solving the Anderson two-impurity model, we will firstly verify no FM phase in the range of parameters investigated under the equilibrium condition in SDQDs. Then, we will report a robust FM phase under nonequilibrium conditions at finite bias and detuning energy, which are strong enough to suppress the AFM phase in the strongly correlated limit $(t\ll U)$. We will demonstrate that the FM exchange interaction origins from the passive parallel spin arrangement caused by the PEP during the electrons transport [see Fig. 1(b)]. At large *t*, the AFM phase keeps stable, which defines a tunnel-barrier control of spin states through the FM–AFM transition in SDQDs, similar to the initial proposal in ref.^18^ but no magnetic field (or auxiliary FM-dots) needed any more.

The FM phase is the effect of PEP on magnetic order, which shows different properties from another effect of PEP called Pauli spin blockade (PSB). The PSB was first observed in vertically coupled GaAs/AlGaAs double quantum dots in 2002^19^, and then received extensive experimental and theoretical studies in various quantum dot systems^20–24^. Fundamentally, the hopping of electrons between two dots can be influenced by their spin configuration. When the total excess electrons of the SDQD is *N*_*T*_ = *N*_1_ + *N*_2_ = 2 with occupation state (*N*_1_, *N*_2_) = (2, 0), (1, 1) or (0, 2), the probability of formation of the spin triplet state *T*(1, 1) may be much larger than that of singlet *S*(1, 1) under one direction of bias of voltage, and then the transport of electrons is blocked due to the PEP which is unfavor of *T*(0, 2) or *T*(2, 0). The story does not happen under the other direction of bias. As a consequence, the current–voltage (*I* − *V*) curve will show a rectification behaviour. As an effect of the PEP on electric current, the PSB is mainly measured and manipulated in the boundary of Coulumb blockage (CB)^20,24^. Basing on previous results of PSB in literatures, we would like to discuss the following two issues: what is the effect of PEP on magnetic order? and what happens in the deep CB area?

## FM phase in SDQDs

The SDQDs we study here can be described by the nonequilibrium Anderson two-impurity model. The total Hamiltonian reads *H*_t*otal*_ = *H*_S_ + *H*_res_ + *H*_sys−res_, where the isolated QD part is

1 $${H}_{S}=\sum _{i,s}{\varepsilon }_{i,s}{\hat{c}}_{i,s}^{\dagger }{\hat{c}}_{i,s}+\frac{U}{2}\sum _{i,s}{\hat{n}}_{i,s}{\hat{n}}_{i\bar{s}}+t\sum _{s}({\hat{c}}_{1,s}^{\dagger }{\hat{c}}_{2,s}+{\rm{h}}.{\rm{c}}.),$$

here ${\hat{c}}_{i,s}^{\dagger }$ (${\hat{c}}_{i,s}$) is the operator that creates (annihilates) an *s*-spin (*s* = ↑, ↓) electron with energy ∈_*i*,*s*_ in the dot *i* (*i* = 1, 2). ${\hat{n}}_{i,s}={\hat{c}}_{i,s}^{\dagger }{\hat{c}}_{i,s}$ corresponds to the *s*-spin electron number operator of dot *i*. As mentioned above, *U* (*U* = *U*_1_ = *U*_2_) is the on-dot Coulomb interaction between *s*- and $\bar{s}$-spin electrons ($\bar{s}$ being the opposite spin of *s*), and *t* is the interdot coupling strength.

The Hamiltonians of reservoirs are ${H}_{res}={\sum }_{\alpha ks}({\varepsilon }_{\alpha ks}+{\mu }_{\alpha }){\hat{c}}_{\alpha ks}^{\dagger }{\hat{c}}_{\alpha ks}$, *α* = L, *R*, under the bias *V* = (*μ*_L_ − *μ*_R_)/*e*, where ${\hat{c}}_{\alpha ks}^{\dagger }$ $({\hat{c}}_{\alpha ks})$ denotes the creation (annihilation) operator of an electron in the *s*-spin state in the *α*-reservoir with wave vector *k*. We set the Fermi energy ${E}_{{\rm{F}}}={\mu }_{{\rm{L}}}^{{\rm{e}}q}={\mu }_{{\rm{R}}}^{{\rm{e}}q}=0$ at equilibrium and *μ*_L_/*e* =− *μ*_R_/*e* = *V*/2 at nonequilibrium. The system-reservoir coupling is ${H}_{sys-res}={\sum }_{\alpha kis}{t}_{\alpha kis}{\hat{c}}_{is}^{\dagger }{\hat{c}}_{\alpha ks}+h.c.$ The hybridization function is assumed to be a Lorentzian form ${J}_{\alpha is}(\omega )=\pi {\sum }_{k}{t}_{\alpha kis}{t}_{\alpha kis}^{\ast }\delta (\omega -{\varepsilon }_{\alpha ks})={\rm{\Gamma }}{W}^{2}/({\omega }^{2}+{W}^{2})$.

We adopt the hierarchical equations of motion (HEOM) approach^13,25^ to numerically solve the nonequilibrium Anderson two-impurity model in a nonperturbative fashion. The HEOM can achieve the same level of accuracy as the latest high-level numerical renormalization group (NRG)^26^ for both static and dynamical quantities under equilibrium conditions^13^. Under nonequilibrium conditions, the HEOM has many advantages above other approaches in the prediction of dynamical properties^24,27–32^. The details of the HEOM formalism and the derivation of physical quantities are supplied in ref.^13,25,33^. The parameters in our calculations are chosen as follows: the on-dot *e* − *e* interaction *U* = 2.0 meV; the singly occupied energy level *∈*_1↓_ = *∈*_1↑_ = − 1.0 meV + Δ and ∈_2↓_ = *∈*_2↑_ = −1.0 meV − Δ, where the detuning energy 2Δ can be finely regulated by gate voltages in experiments; the temperature *T* = 0.1 meV unless otherwise noted; the effective bandwidth of the reservoirs *W*_L_ = *W*_R_ = *W* = 4.0 meV and the reservoir-dot coupling strength Γ_L_ = Γ_R_ = Γ = 0.1 meV. The inter-dot coupling *t*, bias of voltage *V* and detuning energy Δ are three main variables in our calculations.

In order to figure out whether there exists a FM state, we calculate the spin-spin correlation function between QD1 and 2,

2 $${C}_{12}\equiv \langle {\overrightarrow{S}}_{1}\cdot {\overrightarrow{S}}_{2}\rangle -\langle {\overrightarrow{S}}_{1}\rangle \cdot \langle {\overrightarrow{S}}_{2}\rangle ,$$

where ${\overrightarrow{S}}_{i}$ is the quantum spin operator at dot *i*. In Fig. 1(c)–(e), we depict the phase diagram at bias *V* = 0, 0.5 and 1.0 mV, characterized by the sign and value of *C*_12_ in the Δ − *t* plane. Under the equilibrium condition, as shown in Fig. 1(c), the sign of *C*_12_ keeps always negative, which indicates a single AFM phase independent of *t* (*t* > 0) and Δ. It is understandable. From the second-order perturbation, one can obtain ${J}_{{\rm{AF}}}\sim 4{t}^{2}U/[{U}^{2}-{\mathrm{(2}{\rm{\Delta }})}^{2}]$ at finite Δ, seeming a negative *J*_AF_ included. However, the condition for that equation ($t\ll U$ and Δ < *U*/2) makes *J*_*AF*_ < 0 impossible, even under nonequilibrium conditions. Thus, the following FM phase can not result from this mechanism. As shown in Fig. 1(c), with increasing *t*, *C*_12_ positively increases, and finally an AFM QD-molecule forms in the large *t* limit^34^, as an analogue of hydrogen molecule.

When a positive bias applied, as shown in Fig. 1(d) and (e), our results reveal a FM phase appearing in the region of $0 < t\ll U$ and 0.2*U* < Δ < 0.7*U*. In view of the phase changes from Fig. 1(d,e), the FM phase can be seen as growing from the AFM background at finite bias. The FM–AFM phase boundary (where *C*_12_ changing its sign) seems quite smooth with no abrupt phase transition occurring, instead, a continuous crossover behaviour is clearly visible. With increasing bias, the area of FM phase is enlarged and the strength of exchange interaction enhanced, as *C*_12_ positively increases. In the strongly correlated limit $(0 < t\ll U)$, the FM phase can well suppress the AFM one and dominate the phase diagram at finite *V* and Δ, as shown in Fig. 1(e). However, the AFM molecular state will survive at large *t* and very small Δ, which respectively determine the right and bottom boundary of FM phase. If Δ is too large to destroy the single occupation of any dot, *C*_12_ will decrease to zero rapidly, which determines the upper boundary. The left boundary is naturally at *t* ~ 0. As a comprehensive result, the FM phase forms a closed irregular circle area in the phase diagram, as shown in Fig. 1(d) and (e).

**Figure 2:**
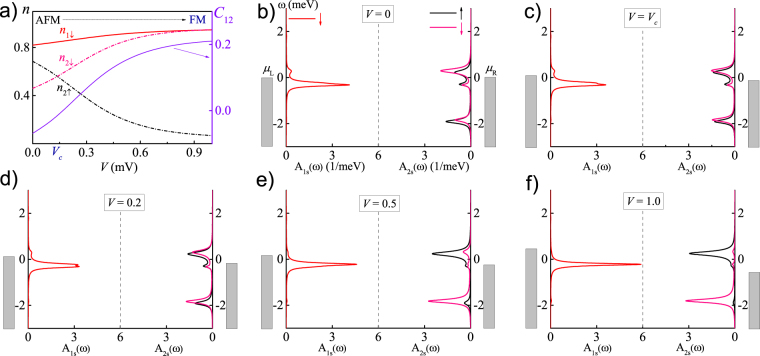
In the case of spin non-degeneracy in QD1 at *t* = 0.2 meV and Δ = 0.75 meV. (**a**) The dependence of *n*_1↓_, *n*_2↑_, *n*_2↓_ and *C*_12_ on *V*. *V*_*c*_ = 0.14 mV is the AFM–FM phase crossover point. (**b**–**f**) The spectral functions *A*_1↓_ (*ω*), *A*_2↑_ (*ω*) and *A*_2↓_ (*ω*) at (**b**) *V* = 0; (**c**) *V* = *V*_*c*_; (**d**) *V* = 0.2 mV; (**e**) *V* = 0.5 mV; and (**f**) *V* = 1.0 mV. The unit of *V* in the figure is mV.

In order to better understand the details of the AFM–FM transition, we theoretically lift the spin degeneracy in QD1 by applying a local magnetic field *B*_1_, with its direction paralleling to ↓-spins. *B*_1_ is chosen to be strong enough to push *∈*_1↑_ much higher than *μ*_L_ but left *∈*_1↓_ = −1.0 meV + Δ, which can be achieved by simultaneously adjusting the gate voltage on QD1. By fixing *t* = 0.2 meV and Δ = 0.75 meV, we calculate both static and dynamical quantities as functions of *V* and summarize the results in Fig. 2, where Fig. 2(a) depicts some typical static quantities (*n*_1↓_, *n*_2↑_, *n*_2↓_ and *C*_12_) and Fig. 2(b)–(e) show the spectral functions [*A*_1↓_ (*ω*), *A*_2↑_ (*ω*) and *A*_2↓_ (*ω*)] at *V* = 0, 0.14 (*V*_*c*_, AFM–FM phase crossover point), 0.2, 0.5, 1.0 mV, respectively. As a starting point, the AFM phase at *V* = 0 is clearly shown in Fig. 2(a), where the magnetic moments *m*_1_ ≡ *n*_1↑_ − *n*_1↓_ ≈ −*n*_1↓_ < 0 and *m*_2_ ≡ *n*_2↑_ − *n*_2↓_ > 0. Accordingly, the degeneracy of *A*_2↑_ (*ω*) and *A*_2↓_ (*ω*) is lifted due to the AFM exchange interaction *J*_AF_, as shown in Fig. 2(b), where the singly-occupation transition peak of *A*_2↑_ (*ω*) is higher than that of *A*_2↓_(*ω*).

Under nonequilibrium conditions,  _↓_ -spin electrons irreversibly flow from L- to R-reservoir through interdot tunneling. During the transport process, the PEP affects both electrical^19,23,24^ and magnetic properties, of which the latter is our focus here. In Fig. 2(a), the continuous crossover from AFM to FM phase is shown in detail. With increasing *V*, *n*_2↑_ gradually decreases while *n*_2↓_ increases, thus *m*_2_ positively decreases. At *V* ~ 0.14 mV, ${n}_{2\uparrow }={n}_{2\downarrow }\Rightarrow {m}_{2}\sim 0$. As a consequence, *C*_12_ ~ 0, which defines an AFM–FM phase crossover point, *V*_*c*_, as shown in Fig. 2(a). By checking the spectral functions, we find the singly-occupation transition peak of *A*_2↑_ (*ω*) almost overlaps with that of *A*_2↓_ (*ω*) at *V* = *V*_*c*_ with a little splitting [see Fig. 2(c)]. With further increasing *V* at *V* > *V*_*c*_, *m*_2_ becomes to negatively increase and *C*_12_ positively increase, as shown in Fig. 2(a), thus the FM phase is gradually enhanced. At *V* ~ 0.9 mV, both *m*_2_ and *C*_12_ reach their saturation values of 0.9 and 0.21, respectively. The continuous increase of *C*_12_ with a smooth sign change indicates the competition between AFM and FM phases is far from intense.

Fundamentally, finite bias injects _↓_-spin electrons from L-reservoir into QD1, followed by interdot tunneling to QD2. In the next step, the PEP prohibits the double occupation of two _↓_-spin electrons, and electrons can only flow out through off-resonance cotunneling^35^ or many-body tunneling^29^ into R-reservoir, both of which produce small current. As shown in Fig. 1(b), for electrons in QD2, increasing *V* and/or Δ will enhance their inflowing probability and meanwhile decrease their off-resonance outflowing probability. When the former becomes much larger than the latter at *V* > *V*_*c*_ and Δ > 0.2*U*, _↓_-spin electrons will accumulate within QD2, which induces a positive to negative sign change of *m*_2_. As a consequence, the exchange of ${\overrightarrow{S}}_{2}$ produces a FM order characterized by *C*_12_ > 0. The spectral functions shown in Fig. 2(d) at *V* = 0.2 mV verifies this FM correlation (although still weak), where the singly-occupation transition peak of *A*_2↑_ (*ω*) becomes lower than *A*_2↓_ (*ω*).

With further increasing *V*, the FM exchange interaction becomes stronger. In spectral functions, this trend is represented by the gradually increasing of the singly-occupation transition peak of *A*_2↓_ (*ω*) and decreasing of that of *A*_2↑_ (*ω*) [see Fig. 2(e)]. At *V* > 0.9 mV, the former reaches its maximum value and the latter almost disappears, as shown in Fig. 2(f). By summarizing Fig. 2(a–f), one can see that the FM phase in SDQDs origins from the passive parallel spin arrangement caused by the PEP during the electrons transport in the presence of *e* − *e* interactions. That mechanism is universal, which should play roles in other strongly correlated models including the Hubbard model.

## Low temperature properties

**Figure 3:**
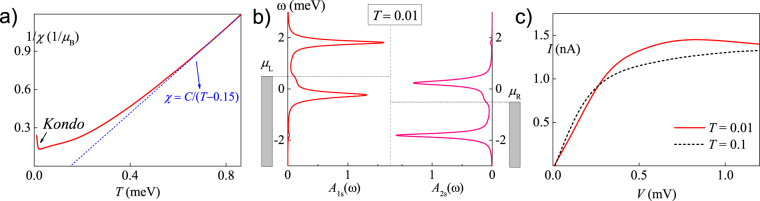
In the case of spin degeneracy in both QDs at *V* = 1.0 mV, *t* = 0.2 meV and Δ = 0.75 meV. (**a**) The dependence of the inverse of magnetic susceptibility 1/*χ* on temperature *T*. The dash line is the fitting of the Curie-Weiss law at high temperature. A Kondo screening effect is shown at *T* < 0.02 meV. (**b**) The spectral functions *A*_*is*_(*ω*) s at temperature below (*T* = 0.01 meV) the Kondo temperature. The current-voltage (*I* − *V*) curves at temperature below (*T* = 0.01 meV, the solid line) and above (*T* = 0.1 meV, the dashed line) the Kondo temperature. The unit of *T* in the figure is meV.

We are now on the position to elucidate the temperature effect, especially the low temperature properties of the FM phase. In what follows, we recover the spin degeneracy in QD1 and fix *V* = 1.0 mV, *t* = 0.2 meV and Δ = 0.75 meV. The dependence of the inverse of magnetic susceptibility 1/*χ* on temperature *T* is depicted in Fig. 3(a), which shows an unambiguous Curie-Weiss behaviour at high temperature, *χ* = *C*/(*T* −*T*_*c*_), with a fitted Curie point *T*_*c*_ ~ 0.15 meV (~1.75 K). We also find a upward deviation at very low temperature *T* < 0.02 meV, resulting from the Kondo screening of the FM phase at *T* < *T*_K_. Under equilibrium conditions, this kind of *S* = 1 Kondo screening induces a ‘singular Fermi liquid state’^36–38^. Here, some nonequilibrium Kondo features are expected.

The present HEOM approach can not directly determine *T*_K_ as NRG does, but it can easily obtain spectral functions and current at sufficient low temperature to elucidate nonequilibrium Kondo characteristics. The HEOM results of *A*_*is*_(*ω*) s and current-voltage (*I* − *V*) curve at *T* = 0.01 meV are respectively shown in Fig. 3(c) and (d), where the *I* − *V* curve at *T* = 0.1 meV (*T* > *T*_K_) is also shown for comparison. As shown in Fig. 3(b), one small Kondo peak is developed at *ω* = *μ*_L_ in *A*_1*s*_(*ω*), and another developed at *ω* = *μ*_R_ in *A*_2*s*_(*ω*). It can be seen as the DQD extension of the bias-induced Kondo peak splitting in single QDs^39^. Although the Kondo peaks in *A*_*is*_(*ω*) s seem not high in Fig. 2(b), their effects are quite significant on both of the magnetic and transport properties. For the latter, the nonequilibrium Kondo resonance assists the electrons transport, which is characterized by the low-temperature current enhancement shown in Fig. 3(c), when the FM phase dominates at *V* > 0.25 mV.

## FM phase in stability diagrams

**Figure 4:**
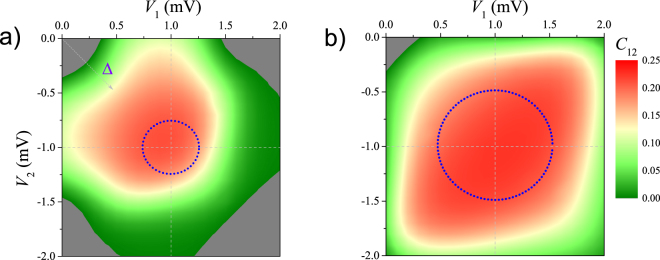
Magnetic phase diagrams of SDQDs in the *V*_1_ − *V*_2_ plane (*V*_1_/*V*_2_ being the gate voltage applied onto QD1/QD2) by showing *C*_12_ at (**a**) *V* = 0.5 mV, *t* = 0.15 meV; and (**b**) *V* = 1.0 mV, *t* = 0.25 meV. The AFM phase is shown in the colour of dark gray. The dashed gray arrow indicates the increasing direction of Δ, and the dashed gray lines schematically mark off the boundary of Coulumb blockage (or degenerate lines in stability diagrams). The dotted blue circle (with the radius of *V*/2) approximately shows the range of PSB.

We can further elucidate the effect of PEP on magnetic order in stability diagrams by expanding the parameter Δ to the *V*_1_ − *V*_2_ plane, where *V*_1_/*V*_2_ is the gate voltage applied onto QD1/QD2. It will help us to directly compare the parameter regions of FM state and PSB. The results at (*V*, *t*) = (0.5 mV, 0.15 meV) and (*V*, *t*) = (1.0 mV, 0.25 meV) are summarized in Fig. 4(a) and (b), respectively. In the figure, the AFM phase is shown in the colour of dark gray, and the dashed gray lines schematically mark off the boundary of CB (or degenerate lines in stability diagrams). By referring the figure, one can see that the FM phase expands into the deep CB area. At *V* = 1.0 mV, as shown in Fig. 4(b), the FM phase occupies almost all of the stability diagram of 0 ≤ *V*_1_ ≤ 2.0 mV(+*U*) and −2.0 mV (−*U*) ≤ *V*_2_ ≤ 0. Basing on experimental observations and theoretical results in literatures^19,20,24^, the range of PSB is approximately within the dotted blue circle (with the radius of *V*/2) in Fig. 4. Obviously, the range of FM phase is much larger than that of PSB.

Taking the case of *V* = 0.5 mV as an example, as shown in Fig. 1(a), if we start from the center of (1, 1) occupation state (*V*_1_ = *V*_2_ = 0), we will first reach a weak FM phase after a AFM–FM transition at *V*_1_(*V*_2_) ~ 0.3 mV. Then, we will follow the enhancement of FM phase with *C*_12_ gradually increasing. Only when *V*_1_(*V*_2_) ~ 0.75 mV, we can observe the PSB effect. It thus indicates that the effect of PEP on magnetic order (FM phase) is prior to that on electric current (PSB). Our HEOM calculations have precisely captured the main features of the former.

## Summary

In summary, we have theoretically reported a robust ferromagnetic phase under nonequilibrium conditions in series-coupled double quantum dots by nonperturbatively solving the Anderson two-impurity model. The ferromagnetic exchange interaction origins from the passive parallel spin arrangement caused by the Pauli exclusion principle during the electrons transport. The ferromagnetic phase can conduce to understand the Heisenberg’s initial idea of ferromagnetic order. In addition, it also predicts a convenient way to internally control spin states without magnetic field.

## Method

The serially coupled DQD system constitutes the open system of primary interest, and the surrounding reservoirs of itinerant electrons are treated as environment. The total Hamiltonian for the system is *H*_t*otal*_ = *H*_S_ + *H*_res_ + *H*_sys-res_, where the interacting DQD

3 $${H}_{S}=\sum _{i,s}{\varepsilon }_{i,s}{\hat{c}}_{i,s}^{\dagger }{\hat{c}}_{i,s}+\frac{U}{2}\sum _{i,s}{\hat{n}}_{i,s}{\hat{n}}_{i\bar{s}}+t\sum _{s}({\hat{c}}_{1,s}^{\dagger }{\hat{c}}_{2,s}+{\rm{h}}.{\rm{c}}.),$$

here ${\hat{c}}_{i,s}^{\dagger }$ (${\hat{c}}_{i,s}$) is the operator that creates (annihilates) a spin-*s* electron with energy *∈*_*i*,*s*_ (*i* = 1, 2) in the dot *i*. ${\hat{n}}_{i,s}={\hat{c}}_{i,s}^{\dagger }{\hat{c}}_{i,s}$ corresponds to the operator for the electron number of dot *i*. *U* (*U* = *U*_1_ = *U*_2_) is the on-dot Coulomb interaction between electrons with spin *s* and $\bar{s}$ (opposite spin of *s*), while *t* is the interdot coupling strengths between the dot 1 and 2, determined by overlapping integral of them.

In what follows, the symbol *μ* is adopted to denote the electron orbital (including spin, space, *etc*.) in the system for brevity, i.e., *μ* = {*s*, *i*...}. The device leads are treated as noninteracting electron reservoirs and the Hamiltonian can be written as

4 $${H}_{{\rm{res}}}=\sum _{\alpha ks}({\varepsilon }_{\alpha ks}+{\mu }_{\alpha }){\hat{c}}_{\alpha ks}^{\dagger }{\hat{c}}_{\alpha ks},\,\alpha ={\rm{L}},{\rm{R}},$$

and the term of dot-electrode coupling is

5 $${H}_{{\rm{sys}}-{\rm{res}}}=\sum _{\alpha kis}{t}_{\alpha kis}{\hat{c}}_{is}^{\dagger }{\hat{c}}_{\alpha ks}+{\rm{h}}\mathrm{.}{\rm{c}}\mathrm{.,}$$

where *∈*_*kα*_ is the energy of an electron with wave vector *k* in the *α* lead, and ${\hat{c}}_{k\mu \alpha }^{\dagger }$ $({\hat{c}}_{k\mu \alpha })$ corresponds to the creation (annihilation) operator for an electron with the *α*-reservoir state |*k*〉 of energy *∈*_*kα*_. To describe the stochastic nature of the transfer coupling, it can be written in the reservoir *H*_*B*_-interaction picture as ${H}_{{\rm{sys}}-{\rm{res}}}={\sum }_{\mu }[{\hat{f}}_{\mu }^{\dagger }(t){\hat{c}}_{\mu }+{\hat{c}}_{\mu }^{\dagger }\,{\hat{f}}_{\mu }(t)]$, with ${\hat{f}}_{\mu }^{\dagger }={e}^{i{H}_{{\rm{res}}}t}[{\sum }_{k\alpha }{t}_{k\mu \alpha }^{\ast }{\hat{c}}_{k\mu \alpha }^{\dagger }]{e}^{-i{H}_{{\rm{res}}}t}$ being the stochastic interactional operator and satisfying the Gauss statistics. Here, *t*_*kμα*_ denotes the transfer coupling matrix element. The influence of electron reservoirs on the dots is taken into account through the hybridization functions, which is assumed Lorentzian form, ${{\rm{\Delta }}}_{\alpha }(\omega )\equiv \pi {\sum }_{k}{t}_{\alpha k\mu }{t}_{\alpha k\mu }^{\ast }\delta (\omega -{\varepsilon }_{k\alpha })={\rm{\Gamma }}{W}^{2}\mathrm{/[2(}\omega -{\mu }_{\alpha }{)}^{2}+{W}^{2}]$, where Γ is the effective quantum dot-electrode coupling strength, *W* is the effective band width, and *μ*_*α*_ is the chemical potentials of the *α* electrode.

In this paper, a hierarchical equations of motion approach (HEOM) developed in recent years is employed to study QD system^13,25^. The HEOM based numerical approach is potentially useful for addressing the interacting strong correlation systems and has been employed to study dynamic properties, such as the dynamic Coulomb blockade Kondo, dynamic Kondo memory phenomena and time-dependent transport with Kondo resonance in QDs systems. The resulting hierarchical equations of motion formalism are in principle exact and applicable to arbitrary electronic systems, including Coulomb interactions, under the influence of arbitrary time-dependent applied bias voltage and external fields. The outstanding issue of characterizing both equilibrium and nonequilibrium properties of a general open quantum system are referred to in ref.^33^. It is essential to adopt appropriate truncated level to close the coupled equations. The numerical results are considered to be quantitatively accurate with increasing truncated level and converge.

Let us make some comments on parameters in our calculations. Most of them are chosen by reference to the typical experiments in DQDs. The on-dot *e-e* interaction and interdot coupling are chosen in the same order as the charging energy and tunnel coupling energy in experiments, and other parameters are flexible within reasonable ranges. However, the band width *W* = *W*_*L*_ = *W*_*R*_ of electrodes is an exception, which is chosen as an effective value only involving those states near equilibrium Fermi energy (and nonequilibrium chemical potentials of electrodes) within the range of [−*W*, *W*]. Although *W* may be as large as several eV for normal metals, a finite effective band width is reasonable if those states out of [−*W*, *W*] plays no role in our results. By checking the effect of *W*, we verify that *W* = 4 meV has fulfilled this condition already.
